# Response to: Reflections on ‘Have we lost sleep?’

**DOI:** 10.1017/mdh.2024.18

**Published:** 2024-07

**Authors:** Niall Patrick Boyce

**Affiliations:** School of Creative Arts, Culture and Communication, Birkbeck University of London, London, United Kingdom of Great Britain and Northern Ireland

**Keywords:** sleep, renaissance, early modern, night, segmented sleep, pre-industrial

## Abstract

I would like to thank Professor Ekirch for his reflections on ‘Have we lost sleep?’, which contain several points that I have already responded to within the paper following his peer review of my original submission to *Medical History* in 2023 (Professor Ekirch having voluntarily identified himself as a reviewer in a normally double-blind process). I acknowledge that the focus of my paper was on Ekirch’s original work from 2001; if I did not engage as he would have wished with his subsequent publications, this was simply because I do not perceive the same substantial developments in his thinking and research on the subject that he does. Indeed, the present critique by Ekirch amounts essentially to more of the same: a long list of references and quotes but little detailed discussion of any individual source. As my paper demonstrates, seemingly unambiguous evidence from a brief quotation can become less clear-cut when placed in context. I am sorry if I deploy the word ‘might’ more than Ekirch would like. This reflects, I hope, a healthy degree of uncertainty and intellectual humility in my approach to the complex issue of pre-industrial sleep. To extend Ekirch’s metaphor, if the jigsaw puzzle that both he and I are trying to assemble can take the form of a cat or a dog, it is possible that its true form is neither animal. The extent to which people woke in the night in pre-industrial Europe, the duration of such awakening, and the predominant cultural attitude towards this—concern, acceptance, or indifference—are topics about which it would seem wise to avoid sweeping statements and generalisations, given the relatively long period covered and the social, cultural, and individual diversity that must be taken into consideration. I can only repeat that I think amassing more brief references, and selectively citing relatively small physiological studies and anthropological evidence from global settings, is unlikely to provide much clarity, let alone definitive answers. I welcome Professor Ekirch’s contribution to this discussion as an indication that the question of segmented sleep in early modern Europe is by no means settled but is a matter of ongoing debate.

I would like to thank Professor Ekirch for his reflections on ‘Have we lost sleep?’, which contain several points that I have already responded to within the paper following his peer review of my original submission to *Medical History* in 2023 (Professor Ekirch having voluntarily identified himself as a reviewer in a normally double-blind process).

I acknowledge that the focus of my paper was on Ekirch’s original work from 2001; if I did not engage as he would have wished with his subsequent publications, this was simply because I do not perceive the same substantial developments in his thinking and research on the subject that he does. Indeed, the present critique by Ekirch amounts essentially to more of the same: a long list of references and quotes but little detailed discussion of any individual source. As my paper demonstrates, seemingly unambiguous evidence from a brief quotation can become less clear-cut when placed in context. I am sorry if I deploy the word ‘might’ more than Ekirch would like. This reflects, I hope, a healthy degree of uncertainty and intellectual humility in my approach to the complex issue of pre-industrial sleep. To extend Ekirch’s metaphor, if the jigsaw puzzle that both he and I are trying to assemble can take the form of a cat or a dog, it is possible that its true form is neither animal.

The extent to which people woke in the night in pre-industrial Europe, the duration of such awakening, and the predominant cultural attitude towards this—concern, acceptance, or indifference—are topics about which it would seem wise to avoid sweeping statements and generalisations, given the relatively long period covered and the social, cultural, and individual diversity that must be taken into consideration. I can only repeat that I think amassing more brief references, and selectively citing relatively small physiological studies and anthropological evidence from global settings, is unlikely to provide much clarity, let alone definitive answers.

I welcome Professor Ekirch’s contribution to this discussion as an indication that the question of segmented sleep in early modern Europe is by no means settled but is a matter of ongoing debate.

